# Individualized Responses of Gut Microbiota to Dietary Intervention Modeled in Humanized Mice

**DOI:** 10.1128/mSystems.00098-16

**Published:** 2016-09-06

**Authors:** Samuel A. Smits, Angela Marcobal, Steven Higginbottom, Justin L. Sonnenburg, Purna C. Kashyap

**Affiliations:** a Department of Microbiology and Immunology, Stanford University School of Medicine, Stanford, California, USA; b Department of Gastroenterology and Hepatology, Mayo Clinic, Rochester, Minnesota, USA; University of California San Diego

**Keywords:** function, gastrointestinal, metabolite, obesity, prebiotic, precision

## Abstract

Diet plays an important role in shaping the structure and function of the gut microbiota. The microbes and microbial products in turn can influence various aspects of host physiology. One promising route to affect host function and restore health is by altering the gut microbiome using dietary intervention. The individuality of the microbiome may pose a significant challenge, so we sought to determine how different microbiotas respond to the same dietary intervention in a controlled setting. We modeled gut microbiotas from three healthy donors in germfree mice and defined compositional and functional alteration following a change in dietary microbiota-accessible carbohydrates (MACs). The three gut communities exhibited responses that differed markedly in magnitude and in the composition of microbiota-derived metabolites. Adjustments in community membership did not correspond to the magnitude of changes in the microbial metabolites, highlighting potential challenges in predicting functional responses from compositional data and the need to assess multiple microbiota parameters following dietary interventions.

**IMPORTANCE** Dietary modification has long been used empirically to modify symptoms in inflammatory bowel disease, irritable bowel syndrome, and a diverse group of diseases with gastrointestinal symptoms. There is both anecdotal and scientific evidence to suggest that individuals respond quite differently to similar dietary changes, and the highly individualized nature of the gut microbiota makes it a prime candidate for these differences. To overcome the typical confounding factors of human dietary interventions, here we employ ex-germfree mice colonized by microbiotas of three different humans to test how different microbiotas respond to a defined change in carbohydrate content of diet by measuring changes in microbiota composition and function using marker gene-based next-generation sequencing and metabolomics. Our findings suggest that the same diet has very different effects on each microbiota’s membership and function, which may in turn explain interindividual differences in response to a dietary ingredient.

**Author Video**: An author video summary of this article is available.

## Abstract (precis)

Dietary modification has long been used empirically to modify symptoms in inflammatory bowel disease, irritable bowel syndrome, and a diverse group of diseases with gastrointestinal symptoms. There is both anecdotal and scientific evidence to suggest that individuals respond quite differently to similar dietary changes, and the highly individualized nature of the gut microbiota makes it a prime candidate for these differences. To overcome the typical confounding factors of human dietary interventions, here we employ ex-germfree mice colonized by microbiotas of three different humans to test how different microbiotas respond to a defined change in carbohydrate content of diet by measuring changes in microbiota composition and function using marker gene-based next-generation sequencing and metabolomics. Our findings suggest that the same diet has very different effects on each microbiota’s membership and function, which may in turn explain interindividual differences in response to a dietary ingredient.

## OBSERVATION

The role of the gut microbiota in maintaining health and causing disease is now well recognized, and yet the operations of this vital component of our biology and the factors driving its function are poorly understood due to its complexity and individuality. Dietary nutrients can have a significant impact on the abundance of specific microbial taxa (1). Some of the most prevalent resources that support the gut microbiota are microbiota-accessible carbohydrates (MACs), the complex carbohydrate portion of dietary fiber (2) that can be metabolized by gut microbes (3). MACs have proven to be a powerful modulator of the microbiota, and purified forms of these carbohydrates (i.e., prebiotics) are increasingly being investigated for therapeutic potential (4).

Predictably shifting the community structure with dietary interventions (5) may be relevant in alleviating the pathogenesis of symptoms associated with chronic gastrointestinal diseases like irritable bowel syndrome and inflammatory bowel disease, where dietary intolerances are common (6). Such strategies have been used as a common intervention (7), and yet the response rates are highly variable, suggesting that interindividual microbiota differences may contribute to this variability. Recent studies in humans highlight the interindividual responses of microbiota composition following specific dietary interventions (1, 8–11); however, the interpretation of the data is limited by a lack of biological replicates for each microbiota composition. Characterization of gut microbial community function has revealed overall conservation at broad levels of functional categorization (12, 13), although long-term dietary trends like veganism appear to influence the serum metabolome, which partially reflects gut microbiota functionality (10). How a specific change in the nutrient milieu influences the conservation of functionality that accompanies individual-specific compositional changes remains a key question.

Here, we investigate structural and functional responses of different human microbiotas to a single microbiota-accessible carbohydrate using a highly controlled experimental system with gnotobiotic mice. Our previous work established that the diversity and metabolomic signatures of a human gut microbiota can be reconstituted in ex-germfree (ex-GF) humanized mice (14).

All experiments were performed according to the A-PLAC, the Stanford IACUC. GF Swiss Webster mice maintained in gnotobiotic isolators were humanized using human fecal samples from healthy donors as previously described (14). Mice were fed a standard polysaccharide-rich diet (Purina LabDiet 5K67) for the first 4 weeks while allowing the microbial community to equilibrate and then switched to a defined diet containing the common prebiotic fructooligosaccharide (FOS; 10% [wt/vol]; Bio-Serv, NJ) for a period of 10 days (see Fig. S1A in the supplemental material). Fecal samples were collected before and after FOS diet intervention and processed for both 16S rRNA-based community composition analysis and fecal metabolomics.

10.1128/mSystems.00098-16.1 Figure S1  (A) Schematic of the experimental setup. Germfree (GF) Swiss Webster mice were maintained in gnotobiotic isolators and humanized with fecal samples from three healthy human donors (D1, D2, and D3). Frozen feces were thawed by dilution in an equal volume of prereduced phosphate-buffered saline under anaerobic conditions, and 0.2 ml nonsettling material was gavaged into the GF recipient mice. Mice were fed a standard polysaccharide-rich diet (Purina LabDiet 5K67) for the first 4 weeks and then switched to a FOS (10% [wt/vol]) diet for 10 days. Fecal samples were collected before (preFOS) and after (postFOS) dietary intervention. The samples were then prepared for both pyrosequencing and metabolomics. For 16S rRNA-based analysis, after fecal DNA isolation (MoBio fecal DNA kit; Carlsbad, CA), 626-bp amplicons spanning the V3-V5 region of bacterial 16S rRNA were generated using barcoded forward primers (338F and 906R). Samples were sequenced using the Roche 454 Titanium platform (Indianapolis, IN, USA). Postprocessing was performed using QIIME (15). For metabolomic surveys, the samples were extracted by using solid-phase Oasis extraction cartridges (Waters, Milford, MA, USA), and fecal metabolites were eluted with 500 µl methanol followed by reverse-phase liquid chromatography and run on an Exactive Orbitrap mass spectrometer (Thermo, Fisher, Waltham, MA, USA) operated in positive and negative electrospray modes. Data analyses were performed by first identifying and deconvoluting peaks using MZmine, manually removing features that were artifacts, aligning peaks using XCMS as previously described (14), and cropping peaks according to elution gradient. Statistical analyses were performed using Python libraries SciPy, scikit-learn, and R’s Stats Library. (B) Supervised learning (linear discriminant analyses) applied to donor community members differentiates between pre-FOS and post-FOS treatment on LD_1_. Glycoside hydrolase 70 and 64 copy numbers are closely associated with dietary intervention. Box plots are on a log scale. (C) The post-FOS treatment group’s microbiota has markedly higher GH70 (α-1,2-branched dextransucrases and α-4,6-glucanotransferases) gene copy numbers than pre-FOS mice. (D) The post-FOS mice’s microbiota possesses fewer GH64 (β-1,3-glucanase) gene copy numbers than the pre-FOS counterparts. Download Figure S1, PDF file, 1.4 MB. Copyright © 2016 Smits et al. 2016 Smits et al. This content is distributed under the terms of the Creative Commons Attribution 4.0 International license.

Postpyrosequencing (454 Titanium) data analysis using QIIME (15) identified 713 ± 251 unique operational taxonomic units (OTUs) on average per mouse and 208 ± 35 unique OTUs on average per mouse after removing singletons. The microbial communities were allowed to establish themselves stably for 4 weeks based on prior studies (16) prior to a dietary change. Before the dietary intervention, two of the microbiotas (D1 and D2) were similar in composition (*Bacteroides* and *Parabacteroides* constituting more than half), contrasting with that of D3 mice, which was dominated by *Clostridiales*. Furthermore, D3’s phylogenetic alpha-diversity was significantly higher (*P* < 6.3e−07; see Table S1 in the supplemental material).

10.1128/mSystems.00098-16.3 Table S1  Phylogenetic (PD_whole_tree) alpha-diversity between the three donors before and after dietary intervention. Download Table S1, XLSX file, 0.1 MB. Copyright © 2016 Smits et al. 2016 Smits et al. This content is distributed under the terms of the Creative Commons Attribution 4.0 International license.

**FIG 1:**
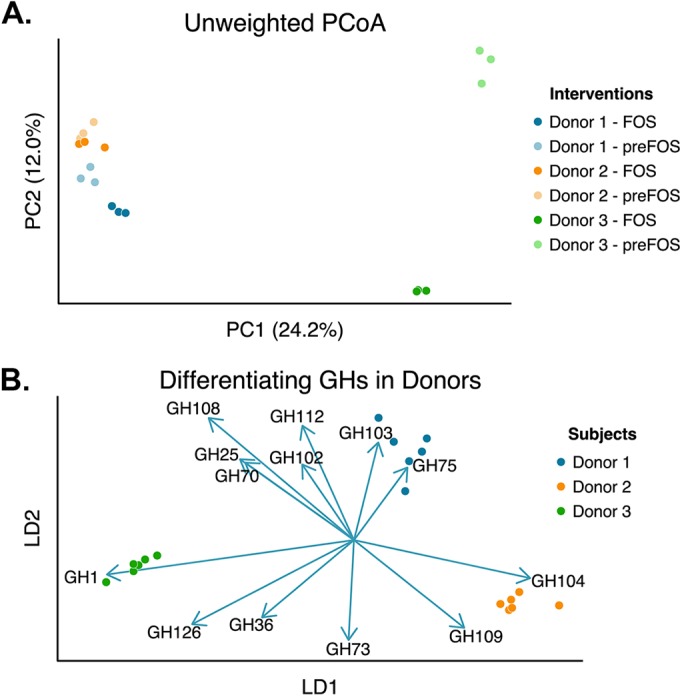
Effect of dietary change on gut microbial community structure. (A) Unweighted UniFrac-based PCoA plot of 16S rRNA profiles showing differences in gut microbiotas in ex-GF mice colonized with three distinct gut microbial communities before and after a change from standard to FOS diet (10% [wt/vol]). (B) Change in glycoside hydrolase (GH) profile using linear discriminant analysis in the three groups of mice following change from standard to FOS diet as imputed from 16S rRNA sequence data.

Following dietary intervention, distinct compositional changes were detected in each microbiota with various magnitudes as revealed by unweighted UniFrac principal coordinate analysis (PCoA) (Fig. 1A). The two similar microbiotas (D1 and D2) exhibited marginal compositional adjustments with respect to PC1 and PC2, in contrast to D3 mice, which showed a marked change along PC2. Taxonomic assignments up to the species level revealed similar traits, with D3 showing the most significant variability in taxonomic composition across the dietary intervention (see Table S2 in the supplemental material). For example, within D3 *Clostridiales* decreased from a mean of 45.6% to 5.8%, while *Allobaculum* increased from a mean of 0% to 31% (*P* < 0.05, Mann-Whitney-Wilcoxon test; Benjamini-Hochberg false discovery rate [FDR] correction). *Bacteroides fragilis*, *Sutterella* species, and *Barnesiellaceae* also increased in D3 while other *Clostridiales*, *Ruminococcus* and *Oscillospira* species, decreased. In contrast, *Lachnospiraceae* (a *Clostridiales* family) increased significantly in D2 (from a mean of 8.8% to 21.9%), accompanied by decreases in *Paraprevotella* species and *Bacteroides ovatus*. The significant changes in D1 were small in terms of magnitude, with increases in *Barnesiellaceae*, *Butyricimonas*, and *Paraprevotella* and decreases in *Clostridiales* (3.9% to 0.1%) and *Coprobacillus* species. The current 16S rRNA-based sequencing technology and available reference databases preclude more precise identification at the species level, and yet the above findings highlight interindividual variability in compositional response among the three groups of humanized mice.

10.1128/mSystems.00098-16.4 Table S2  Statistically significant differentiating taxa categorized to species level between donors. Download Table S2, XLSX file, 0.1 MB. Copyright © 2016 Smits et al. 2016 Smits et al. This content is distributed under the terms of the Creative Commons Attribution 4.0 International license.

We next applied supervised learning approaches to determine the confidence with which individual specific changes could be reliably predicted. Donor-specific changes due to diet were predicted with no detectable error (see Table S3 in the supplemental material) consistent with the individuality of composition and response. FOS diet-related responses across all three groups were predictable with 6 to 22% error (see Tables S4 and S5 and Fig. S1B). Next, given that the dietary intervention involved a significant shift in carbohydrates, we imputed changes in the community’s glycoside hydrolase (GH) functional capacity using 16S rRNA data and a method that intentionally cripples the imputations by overgeneralizing across available reference genomes and thereby increasing confidence in signals that overcome this blurring, as previously validated (17). Glycoside hydrolase families capture various degrees of carbohydrate specificity, with multiple families sometimes representing similar functions, for which linear combination analyses such as linear discriminant analyses (LDA) are appropriate. Interestingly, applying this supervised learning approach to the imputed GH profiles reliably predicted the individual donors (Fig. 1B), consistent with an individual and specific reorganization of functional capacity following FOS diet introduction. Furthermore, GH70 and GH64 were closely associated with the dietary intervention when assessed in donor groups (see Fig. S1C and D).

10.1128/mSystems.00098-16.5 Table S3  OTU identified as important features by supervised learning (random forests) for differentiating between the donors across the FOS dietary intervention. Download Table S3, XLSX file, 0.1 MB. Copyright © 2016 Smits et al. 2016 Smits et al. This content is distributed under the terms of the Creative Commons Attribution 4.0 International license.

10.1128/mSystems.00098-16.6 Table S4  OTU identified as important features by supervised learning (random forests) for differentiating between the FOS dietary intervention samples using donor gut microbiota members. Download Table S4, XLSX file, 0.1 MB. Copyright © 2016 Smits et al. 2016 Smits et al. This content is distributed under the terms of the Creative Commons Attribution 4.0 International license.

10.1128/mSystems.00098-16.7 Table S5  Top 20 OTU scalings identified by supervised learning (linear discriminant analyses) applied to donor gut microbiota members across the FOS dietary intervention. Download Table S5, XLSX file, 0.1 MB. Copyright © 2016 Smits et al. 2016 Smits et al. This content is distributed under the terms of the Creative Commons Attribution 4.0 International license.

Given the specific functional changes, we next performed nontargeted metabolomics using ultraperformance liquid chromatography-mass spectrometry (MS) on the same fecal samples as previously described (14). Briefly, fecal water samples were extracted by using solid-phase Oasis extraction cartridges (Waters, Milford, MA, USA). Chromatographic separation was performed on a 150-mm by 2.1-mm Kinetex 1.7-µm C_18_ column (Phenomenex, Torrance, CA) using an Acquity ultraperformance liquid chromatography system (Waters). The flow rate was 0.25 ml ⋅ min^−1^. The column was held at 40°C. Solvent A was 10 mM ammonium formate in water, and solvent B was 10 mM ammonium formate in methanol. The gradient started at 5% B and linearly increased to 10% B at 14 min and then linearly increased to 100% B at 22 min and was held at 100% B for 5 min. The column was equilibrated at 5% B for 3 min before starting the run. First, 1.3 min of mobile-phase flow was diverted from the ion source into the waste. MS was performed on the Exactive (Thermo, Fisher, Waltham, MA, USA) Orbitrap mass spectrometer operated in positive and negative electrospray mode and controlled by Xcalibur 2.1 software.

Using stringent criteria for identifying features in our metabolomics data (significantly higher than baseline intensities and more than 3E4 arbitrary units [AU] in at least one sample and manually curated for peaks consistent with well-separated compounds), we identified 1,527 total unique features in both electrospray modes. Features meeting the identification criteria were used to identify the same features in other samples that did not meet the intensity criteria after alignment, after which 628 features were identified as common across all samples. We identified 1,475 features shared in at least one sample in each of the dietary groups and 472 that were significantly different (*P* < 0.05; Mann-Whitney-Wilcoxon test) between dietary interventions (see Table S6 in the supplemental material). In contrast, 1,131 features were identified in at least one sample in each of the donors (see Fig. S2A to C)

10.1128/mSystems.00098-16.8 Table S6  Statistically significant differentiating metabolomic features between dietary intervention groups. Four hundred seventy-two metabolomic features across both electrospray modes were identified as being significant (*P* < 0.05, Mann-Whitney-Wilcoxon test) after Benjamini-Hochberg FDR correction for multiple hypotheses. Download Table S6, XLSX file, 0.4 MB. Copyright © 2016 Smits et al. 2016 Smits et al. This content is distributed under the terms of the Creative Commons Attribution 4.0 International license.

**FIG 2:**
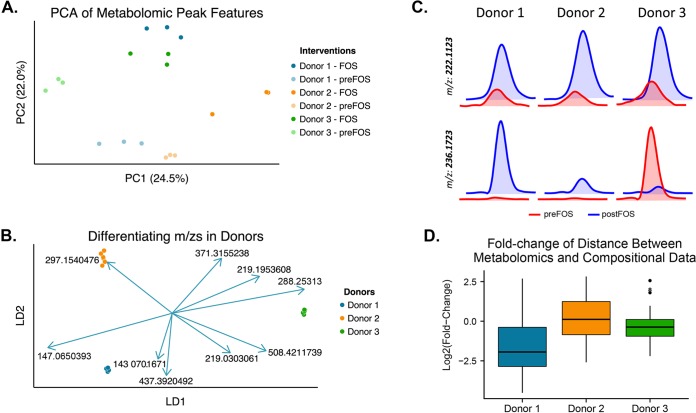
Effect of dietary change on gut microbial function. (A) PCA of metabolomic features detected in feces of ex-GF mice colonized with three distinct human-derived gut microbial communities before and after a change from standard to FOS diet (10% [wt/vol]). (B) Donor-specific metabolites across dietary interventions identified using linear discriminant analysis. The metabolites with the top *m/z* values with various retention times that differentiate the donors are depicted as scaled vectors. (C) Liquid chromatography elution profiles of two metabolites before and after dietary change in the three groups of mice. (D) Fold changes of the distances between metabolomics and compositional data from standard to FOS diet vary significantly (*P* < 0.05; Wilcoxon rank test) across all of the donor groups.

Principal component analysis of the metabolomic features revealed that the magnitude of change following the introduction of a FOS diet did not correspond to the magnitude of change observed in composition (Fig. 2A). In fact, the variances explained between samples in the metabolomics data were only negligibly correlated with those in compositional data (*R* = 0.66, *P* < 0.001; Mantel’s Pearson test, 1,000 permutations). Specific features (*m/z* values) were better predictors of individual donors than the dietary intervention, suggesting individualized functional changes following diet intervention (Fig. 2B; see also Table S7 in the supplemental material). For example, some metabolites such as that with an *m/z* value of 222.1123 were similarly altered in all the groups of mice; other compounds, such as that with an *m/z* value of 236.1723, showed individualized responses (Fig. 2C; also see Table S8 in the supplemental material). Furthermore, after applying a Procrustes transformation which optimally minimized the distances between the metabolomics and compositional data, the fold changes of the distances between fecal samples before and after dietary intervention were significantly different across all groups (Fig. 2D; also see Fig. S2D; P < 0.05, Wilcoxon rank test).

10.1128/mSystems.00098-16.2 Figure S2  Venn diagrams depicting the number of *m/z* features that are distinct and shared across the dietary intervention and all donors. Together, the data show that there are more metabolomic features that are unique to specific donors than to specific dietary conditions. (A) Venn diagram showing *m/z* features shared across dietary interventions (1,475 common, with 46 unique to the FOS-treated group). (B) Venn diagram showing *m/z* features shared across donors. The donors share only 1,131 *m/z* features, highlighting the need to consider the preintervention state of an individual. (C) Venn diagram showing *m/z* features shared (628) across all samples. (D) Euclidean distances between Procrustes-transformed 16S and metabolomics samples within each treatment group show variable responses. Dimensionality reduction methods applied to the 16S and metabolomics data transform the data to a Euclidean space, explaining the variance between samples. We applied a Procrustes transformation to these data to minimize the distances between samples and calculated the Euclidean distances between all pair combinations within the treatment groups, depicted here as box plots. The data show that metabolomics and compositional data for donor 3 were less variable than those for donors 1 and 2. Furthermore, the variances of the distances were not more reduced for a specific dietary treatment; variance was reduced for the pre-FOS group in donors 1 and 3 with respect to the FOS, whereas for donor 2 the variance in the FOS treatment group was reduced with respect to the pre-FOS group. Download Figure S2, PDF file, 0.2 MB. Copyright © 2016 Smits et al. 2016 Smits et al. This content is distributed under the terms of the Creative Commons Attribution 4.0 International license.

10.1128/mSystems.00098-16.9 Table S7  Top identified *m/z* features that drive the differences between donors using linear discriminant analysis. Download Table S7, XLSX file, 0.1 MB. Copyright © 2016 Smits et al. 2016 Smits et al. This content is distributed under the terms of the Creative Commons Attribution 4.0 International license.

10.1128/mSystems.00098-16.10 Table S8  Statistically significant differential changes in metabolites in response to dietary intervention across donors. Download Table S8, XLSX file, 0.1 MB. Copyright © 2016 Smits et al. 2016 Smits et al. This content is distributed under the terms of the Creative Commons Attribution 4.0 International license.

In summary, we describe changes in gut microbiota composition and function from three healthy individuals modeled in ex-GF mice following a defined dietary intervention. Our findings show that compositional changes affected by diet do not necessarily predict community functionality. In the context of precision medicine, our data point to the importance of assessing an individual’s changes in microbiota function in the context of compositional changes following dietary intervention in attempts to rationally manipulate community metabolic output. While this pilot study highlights the differential response to diet in 3 groups of humanized mice, in order to better delineate individualized responses and changes in specific metabolites, larger patient cohorts will need to be investigated with an in-depth profiling of microbial community function. Future studies assessing changes in microbial function following defined dietary interventions in humans will be critical to identify microbiome-encoded determinants of host response to diet.

### Accession number(s).

Postpyrosequencing data were deposited in the NCBI Sequence Read Archive under accession no. SRP080153. Metabolomics data have been deposited in the MassIVE database under accession no. MSV000079922.

## References

[1] Wu GD, Chen J, Hoffmann C, Bittinger K, Chen YY, Keilbaugh SA, Bewtra M, Knights D, Walters WA, Knight R, Sinha R, Gilroy E, Gupta K, Baldassano R, Nessel L, Li H, Bushman FD, Lewis JD 2011 Linking long-term dietary patterns with gut microbial enterotypes. Science 334:105–108. doi:10.1126/science.1208344. 21885731 PMC3368382

[2] Rogowski A, Briggs JA, Mortimer JC, Tryfona T, Terrapon N, Lowe EC, Baslé A, Morland C, Day AM, Zheng H, Rogers TE, Thompson P, Hawkins AR, Yadav MP, Henrissat B, Martens EC, Dupree P, Gilbert HJ, Bolam DN 2015 Glycan complexity dictates microbial resource allocation in the large intestine. Nat Commun 6:7481. doi:10.1038/ncomms8481. 26112186 PMC4491172

[3] Sonnenburg ED, Sonnenburg JL 2014 Starving our microbial self: the deleterious consequences of a diet deficient in microbiota-accessible carbohydrates. Cell Metab 20:779–786. doi:10.1016/j.cmet.2014.07.003. 25156449 PMC4896489

[4] Vulevic J, Juric A, Walton GE, Claus SP, Tzortzis G, Toward RE, Gibson GR 2015 Influence of galacto-oligosaccharide mixture (B-GOS) on gut microbiota, immune parameters and metabonomics in elderly persons. Br J Nutr 114:586–595. doi:10.1017/S0007114515001889. 26218845

[5] David LA, Maurice CF, Carmody RN, Gootenberg DB, Button JE, Wolfe BE, Ling AV, Devlin AS, Varma Y, Fischbach MA, Biddinger SB, Dutton RJ, Turnbaugh PJ 2014 Diet rapidly and reproducibly alters the human gut microbiome. Nature 505:559–563. doi:10.1038/nature12820. 24336217 PMC3957428

[6] Saito YA, Locke GR III, Weaver AL, Zinsmeister AR, Talley NJ 2005 Diet and functional gastrointestinal disorders: a population-based case-control study. Am J Gastroenterol 100:2743–2748. doi:10.1111/j.1572-0241.2005.00288.x. 16393229

[7] Halmos EP, Power VA, Shepherd SJ, Gibson PR, Muir JG 2014 A diet low in FODMAPs reduces symptoms of irritable bowel syndrome. Gastroenterology 146:67–75.e5. doi:10.1053/j.gastro.2013.09.046. 24076059

[8] Salonen A, Lahti L, Salojärvi J, Holtrop G, Korpela K, Duncan SH, Date P, Farquharson F, Johnstone AM, Lobley GE, Louis P, Flint HJ, de Vos WM 2014 Impact of diet and individual variation on intestinal microbiota composition and fermentation products in obese men. ISME J 8:2218–2230. doi:10.1038/ismej.2014.63. 24763370 PMC4992075

[9] Walker AW, Ince J, Duncan SH, Webster LM, Holtrop G, Ze X, Brown D, Stares MD, Scott P, Bergerat A, Louis P, McIntosh F, Johnstone AM, Lobley GE, Parkhill J, Flint HJ 2011 Dominant and diet-responsive groups of bacteria within the human colonic microbiota. ISME J 5:220–230. doi:10.1038/ismej.2010.118. 20686513 PMC3105703

[10] Wu GD, Compher C, Chen EZ, Smith SA, Shah RD, Bittinger K, Chehoud C, Albenberg LG, Nessel L, Gilroy E, Star J, Weljie AM, Flint HJ, Metz DC, Bennett MJ, Li H, Bushman FD, Lewis JD 2016 Comparative metabolomics in vegans and omnivores reveal constraints on diet-dependent gut microbiota metabolite production. Gut 65:63–72. doi:10.1136/gutjnl-2014-308209. 25431456 PMC4583329

[11] Cotillard A, Kennedy SP, Kong LC, Prifti E, Pons N, Le Chatelier E, Almeida M, Quinquis B, Levenez F, Galleron N, Gougis S, Rizkalla S, Batto JM, Renault P, ANR MicroObes Consortium, Doré J, Zucker JD, Clément K, Ehrlich SD 2013 Dietary intervention impact on gut microbial gene richness. Nature 500:585–588. doi:10.1038/nature12480. 23985875

[12] Lozupone CA, Stombaugh JI, Gordon JI, Jansson JK, Knight R 2012 Diversity, stability and resilience of the human gut microbiota. Nature 489:220–230. doi:10.1038/nature11550. 22972295 PMC3577372

[13] The Human Microbiome Project Consortium 2012 Structure, function and diversity of the healthy human microbiome. Nature 486:207–214. doi:10.1038/nature11234. 22699609 PMC3564958

[14] Marcobal A, Kashyap PC, Nelson TA, Aronov PA, Donia MS, Spormann A, Fischbach MA, Sonnenburg JL 2013 A metabolomic view of how the human gut microbiota impacts the host metabolome using humanized and gnotobiotic mice. ISME J 7:1933–1943. doi:10.1038/ismej.2013.89. 23739052 PMC3965317

[15] Caporaso JG, Kuczynski J, Stombaugh J, Bittinger K, Bushman FD, Costello EK, Fierer N, Peña AG, Goodrich JK, Gordon JI, Huttley GA, Kelley ST, Knights D, Koenig JE, Ley RE, Lozupone CA, McDonald D, Muegge BD, Pirrung M, Reeder J, Sevinsky JR, Turnbaugh PJ, Walters WA, Widmann J, Yatsunenko T, Zaneveld J, Knight R 2010 QIIME allows analysis of high-throughput community sequencing data. Nat Methods 7:335–336. doi:10.1038/nmeth.f.303. 20383131 PMC3156573

[16] Turnbaugh PJ, Ridaura VK, Faith JJ, Rey FE, Knight R, Gordon JI 2009 The effect of diet on the human gut microbiome: a metagenomic analysis in humanized gnotobiotic mice. Sci Transl Med 1:6ra14. doi:10.1126/scitranslmed.3000322. PMC2894525 20368178

[17] Kashyap PC, Marcobal A, Ursell LK, Smits SA, Sonnenburg ED, Costello EK, Higginbottom SK, Domino SE, Holmes SP, Relman DA, Knight R, Gordon JI, Sonnenburg JL 2013 Genetically dictated change in host mucus carbohydrate landscape exerts a diet-dependent effect on the gut microbiota. Proc Natl Acad Sci U S A 110:17059–17064. doi:10.1073/pnas.1306070110. 24062455 PMC3800993

