# Supplementary figures and images for: Individualized Responses of Gut Microbiota to Dietary Intervention Modeled in Humanized Mice
Source: mSystems. 2016 Sep 6;1(5):e00098-16. doi: 10.1128/mSystems.00098-16 (PMC5069738; doi:10.1128/mSystems.00098-16)

## A. Schematic of the Experiment

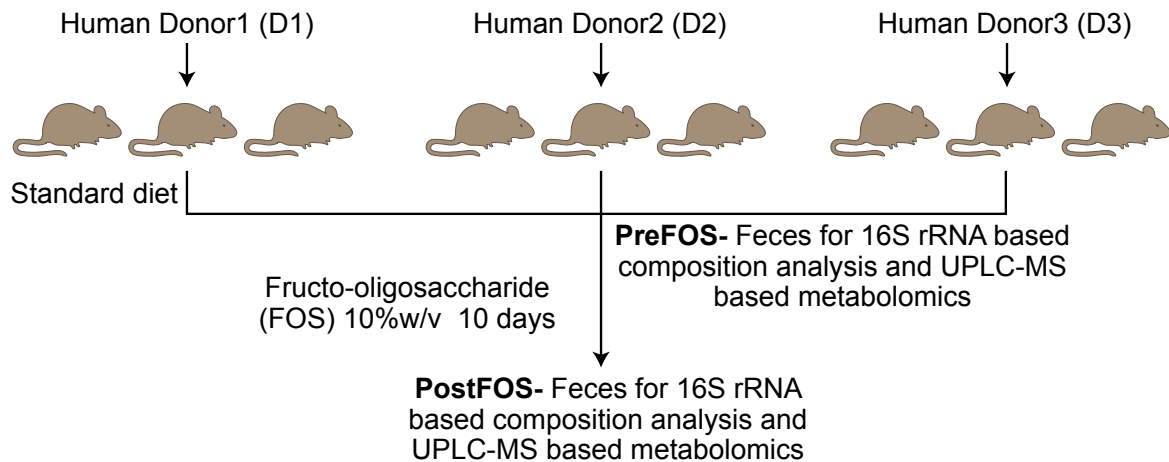

## B.

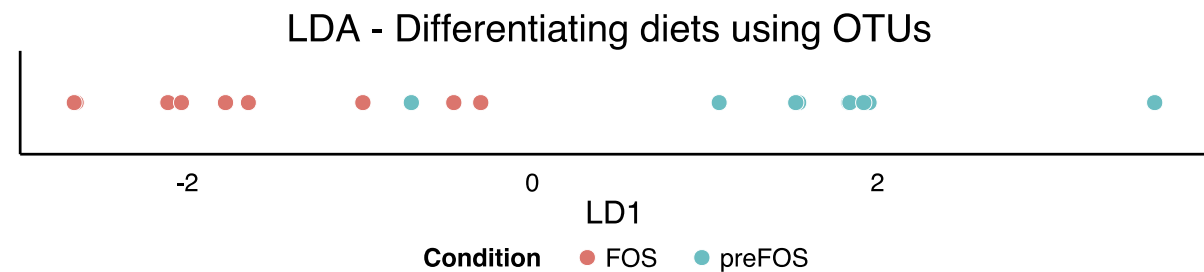

## C.

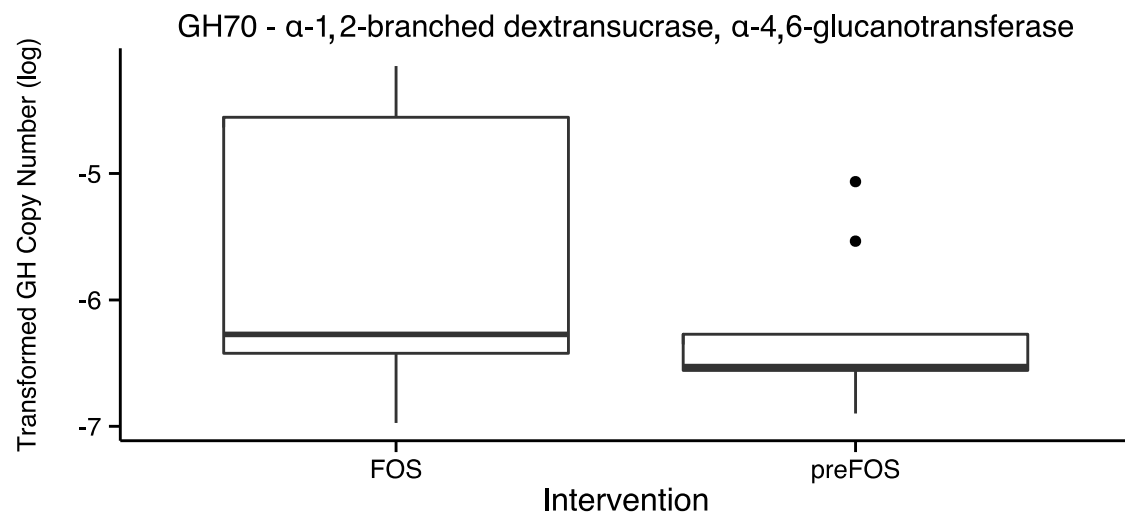

## D.

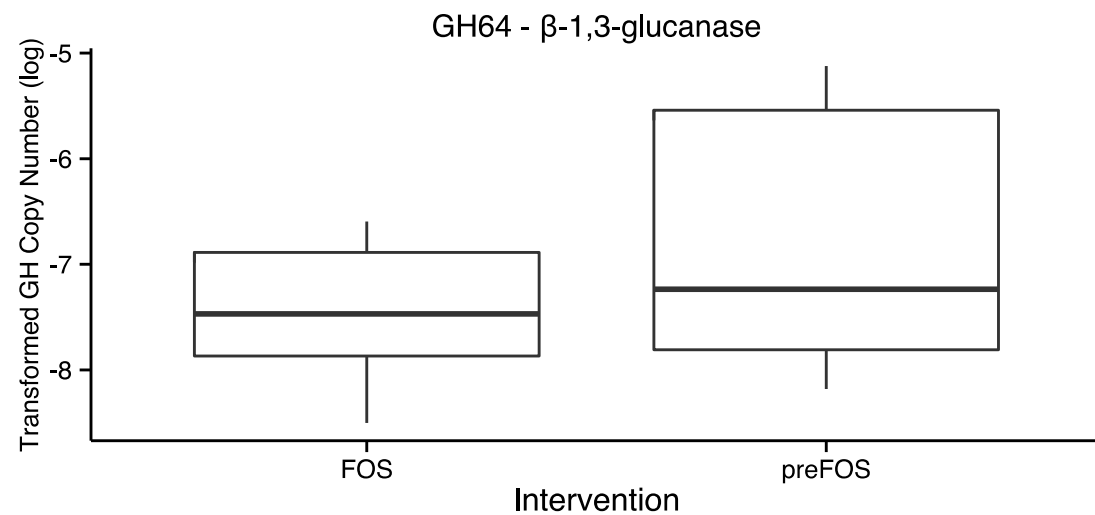

Supplement: Figure S1 [file sys005162051sf1.pdf]

A.

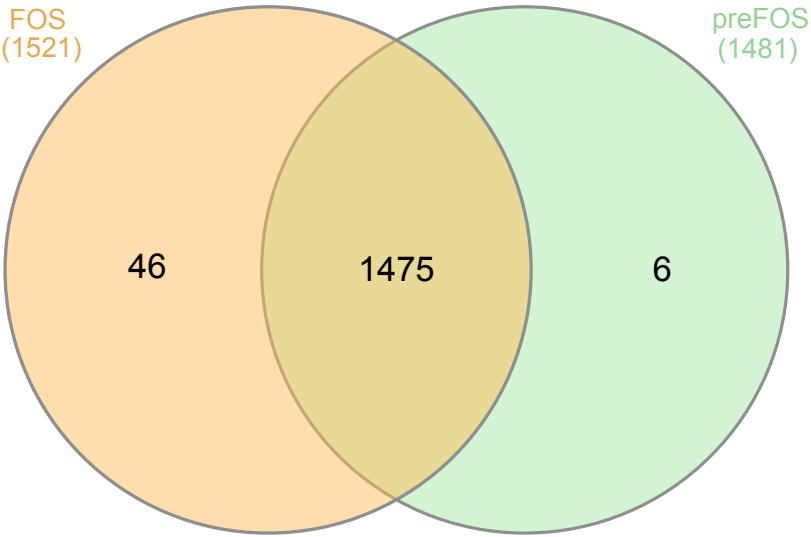

B.

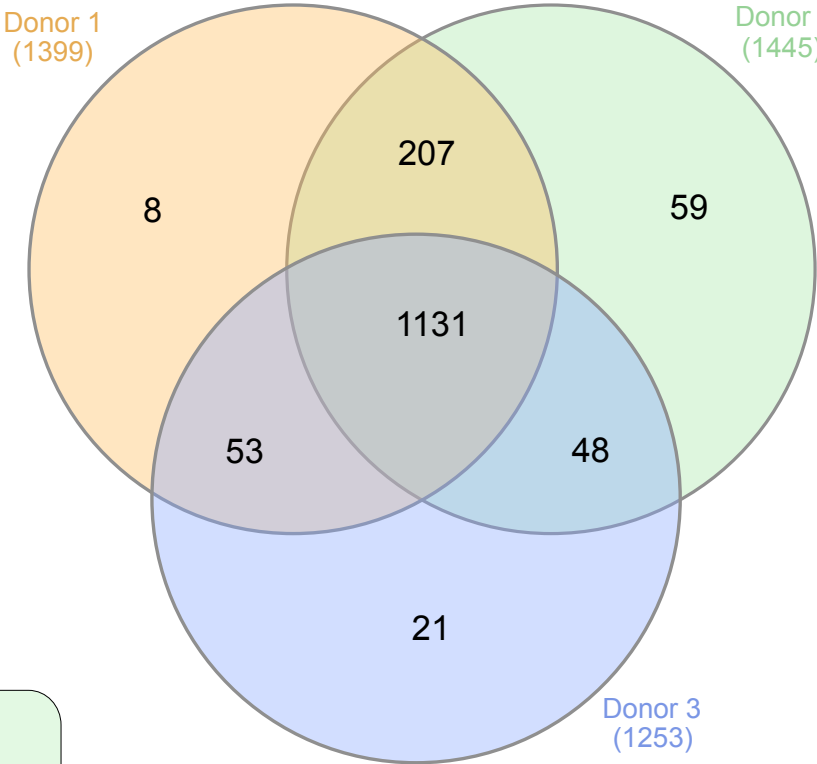

C.

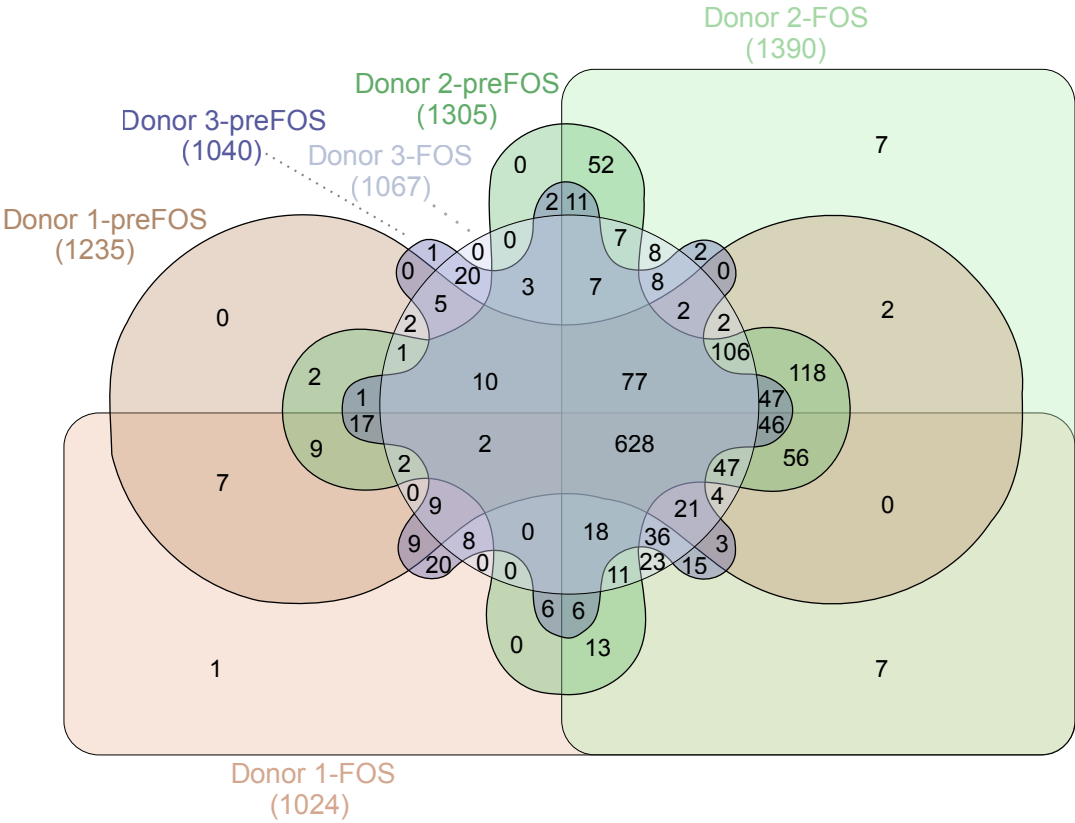

D.

Distance between Metabolomics and Compositional Data

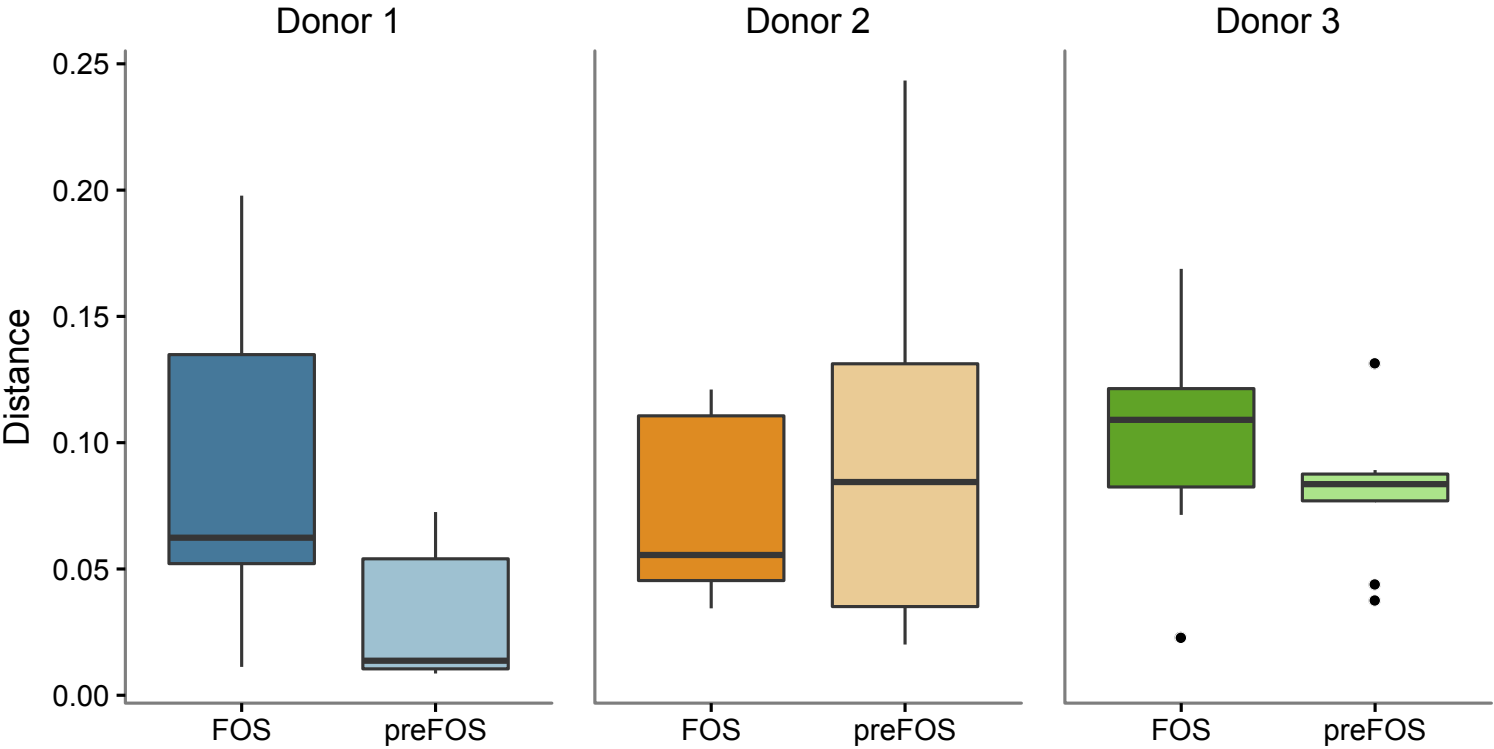

Supplement: Figure S2 [file sys005162051sf2.pdf]
